# Genomic Evolution of *Siccibacter colletis*: Comparative Analysis and First Clinical Isolate Report

**DOI:** 10.3390/microorganisms14040932

**Published:** 2026-04-20

**Authors:** Wentao Zhu, Qian Liu, Xi Chen, Chunxia Yang, Ming Wei, Li Gu, Hui Yuan, Hong Shen

**Affiliations:** 1 Department of Infectious Diseases and Clinical Microbiology, Beijing Chaoyang Hospital, Capital Medical University, Beijing 100020, China; wentaozhu@126.com (W.Z.);; 2 Department of Clinical Laboratory, Beijing Anzhen Hospital, Capital Medical University, Beijing 100029, China

**Keywords:** *Siccibacter*, *Siccibacter colletis*, pathogen identification, comparative genome, bacterial pathogens

## Abstract

The genus *Siccibacter* consists primarily of environmental bacteria, with strains of *Siccibacter colletis* previously isolated only from plant materials and related environments. This study aims to characterize the first clinical isolate of *S. colletis* and explore its genomic evolution and clinical relevance. Strain S25242 was isolated from the urine of a 64-year-old male with a severe urinary tract infection. The genome of S25242 is 4.19 Mb, containing 4012 coding sequences, 73 tRNAs, 10 rRNAs, and 38 snRNAs. Phylogenetic and phylogenomic analyses indicated that strain S25242 is closely related to *S. colletis* type strain 1383^T^. The strain shared >70% of digital DNA-DNA hybridization (dDDH) values and >96% of average nucleotide identity (ANI) values with the type strain of *S. colletis* 1383^T^, thereby confirming its taxonomic status. The isolate was susceptible to all 11 tested antimicrobials. Comparative genomics identified 1942 *S. colletis-specific* genes (including multidrug efflux systems) and 13 unique genes in S25242 related to transposition and DNA integration. This study reports the first clinical isolate of *S. colletis*, providing evidence that genomic plasticity facilitates its transition from an environmental inhabitant to an opportunistic pathogen. The findings highlight the need for enhanced clinical surveillance of the *Siccibacter* genus and offer insights into its genomic evolution and clinical adaptation.

## 1. Introduction

The genus *Siccibacter*, along with its close relatives *Cronobacter* and *Franconibacter*, belongs to the family *Enterobacteriaceae* within the class *Gammaproteobacteria* [1,2,3]. Historically, species within these three genera were classified under the broad genus *Enterobacter*; however, significant taxonomic revisions based on polyphasic evidence have redefined them as distinct monophyletic groups [1]. Despite their close relationship to known pathogens, their genomic profiles reflect diverse metabolic capacities, allowing them to thrive in various habitats ranging from plant material to food production environments [4].

The genus currently includes two species: *Siccibacter turicensis* (the type species, formerly known as *Cronobacter turicensis*) and *Siccibacter colletis* (originally isolated from poppy seeds and tea leaves) [5,6]. *S. turicensis* has been isolated from various foods and has also been described in clinical samples [7]. *S. colletis* is a Gram-negative, motile, and facultatively anaerobic bacterium characterized by its ability to promote plant growth through the production of siderophores and auxin [8]. Genomic analysis of *S. colletis* shows an average nucleotide identity of 87.2% with *S. turicensis*. Furthermore, multilocus sequence analysis (MLSA) of housekeeping genes (*atpD*, *fusA*, *glnS*, *gyrB*, and *infB*) establishes it as a distinct phylogenetic lineage [5].

*Siccibacter* species share environmental, desiccation-resistant characteristics with *Cronobacter* [9], and while primarily environmental, rare instances of clinical isolation (specifically *S. turicensis* from a case of angular cheilitis) highlight a potential, albeit currently limited, pathogenicity [10]. To date, there are only four genomes available for *S. colletis* strains (https://www.ncbi.nlm.nih.gov/datasets/genome/?taxon=1505757, accessed on 26 January 2026), which were isolated from seeds, leaves, and cucumber fermentation brine. *S. colletis* species has not previously been reported in clinical samples. In this study, we identified a clinical isolate in the urine of an inpatient. We further characterized the antimicrobial susceptibility profile and genomic characteristics of this first clinical strain of *S. colletis*. This study is significant for understanding the emerging pathogenicity of this species, underscoring the need for increased attention in future clinical research.

## 2. Materials and Methods

### 2.1. Strain Collection

In this retrospective study, a strain of *Siccibacter* was isolated from the urine of a patient at a tertiary teaching hospital affiliated with Capital Medical University in March 2025. The urine specimen was inoculated onto a China blue agar plate using the three-zones streak method, and incubated at 35 °C for 12 h. The isolate was subsequently identified as *S. colletis* using matrix-assisted laser desorption/ionization time-of-flight MS (MALDI-TOF MS, Bruker, Champs-sur-Marne, France) following previously reported protocols [11,12]. *Escherichia coli* ATCC 8739 was used as the quality control strain.

### 2.2. Genomic Sequencing

To exclude the potential misidentification by MALDI-TOF MS, the isolate was subjected to whole-genome sequencing. Genomic DNA was extracted using the Wizard Genomic DNA extraction kits (Promega, Madison, WI, USA) and sent to Beijing Novogene Biotechnology Co., Ltd. (Beijing, China) for sequencing. The DNA was randomly sheared into short fragments, which were end-repaired, A-tailed, and further ligated with Illumina adapters. The resulting fragments were size-selected, PCR-amplified, and purified. Library quantification was performed using Qubit 2 and real-time PCR, while size distribution was assessed using a bioanalyzer. The library was sequenced on the Illumina Novaseq platform (San Diego, CA, USA). Raw reads were filtered to obtain high-quality data (clean Data) using Trimmomatic v0.40 (https://github.com/usadellab/Trimmomatic (accessed on 16 February 2026)) based on the following criteria: removal of adapters, reads containing >10% undefined nucleotides (N), and reads containing >40% low-quality bases (Q ≤ 20).

### 2.3. Genomic Assembly and Annotation

The obtained clean data was subjected to genome assembly using three software packages, including SOAPdenovo v242, SPAdes v4.2.0, and Abyss v2.3.10, varying K-mers (95, 107, 119) [13,14,15]. The assembly results from these tools were integrated using CISA software (accessed on 16 February 2026) , and the assembly with the fewest scaffolds was selected [16]. Contigs shorter than 500 bp were filtered out, and the final assembly was retained for downstream analysis. The genome was annotated using Rapid Annotation on the Subsystem Technology (RAST) server [17], which classified the genes into 26 subsystem categories. Transfer RNA (tRNA), Ribosome RNA (rRNA), and small non-coding RNAs (sRNAs) genes were predicted by tRNAscan-SE [18], rRNAmmer [19], and BLAST against the Rfam database [20], respectively. The sequence type was determined using the *Cronobacter* spp. multilocus sequence typing (MLST) scheme [21]. Finally, antimicrobial resistance genes (ARGs) were identified using the Comprehensive Antibiotic Resistance Database (CARD) [22]. Virulence factors were identified based on the virulence factor database (VFDB) (https://www.mgc.ac.cn/VFs/) (accessed on 16 February 2026).

### 2.4. Genomic Comparison

Genome-to-genome distances were calculated using digital DNA-DNA hybridization (dDDH) via the “Genome-to-Genome Distance Calculator 3.0” web service [23]. The threshold of species delineation was set as 70%, consistent with the widely accepted gold standard [24]. Pairwise comparisons of whole-genome average nucleotide identity (ANI) were computed using FastANI v1.34 (https://github.com/ParBLiSS/FastANI (accessed on 16 February 2026)), and genomes showing >95% ANI were classified as the same species [25]. Comparative genomics analysis was performed by OrthoVenn3 [26], which identified orthologous gene clusters based on the OrthoMCL algorithm with an e-value of 10^−2^ and an inflation value of 1.50.

### 2.5. Phylogenetic Analysis

Phylogenetic placement and closely related species were initially inferred using the Type (Strain) Genome Server (TYGS) [27]. Genomic comparisons were conducted using GBDP, with intergenomic distances inferred under the ‘trimming’ algorithm and distance formula *d*_5_ [23]. A balanced minimum evolution tree was reconstructed based on the resulting distances using FASTME 2.1.6.1 [28]. Core genes (identified in >95% genomes) from all available *Siccibacter* genomes were extracted using Roary v3.13.0 [29]. A phylogenomic tree based on the core genes was then inferred using FastTree 2 [30] and visualized in Chiplot [31].

### 2.6. Antimicrobial Susceptibility Testing

Antimicrobial susceptibility testing was performed in vitro using the Kirby–Bauer (K-B) disk diffusion method (Oxoid, Basingstoke, UK). The bacterial suspension was diluted to a 0.5 McFarland standard and spread onto Mueller–Hinton (MH) agar plates. Susceptibility disks were then placed on the MH agar, and the plates were incubated at 35 °C for 16–18 h. Eleven antimicrobial agents or combinations were evaluated, including ampicillin (10 μg), cefotaxime (330 μg), ceftriaxone (30 μg), ciprofloxacin (5 μg), gentamicin (10 μg), imipenem (10 μg), levofloxacin (5 μg), meropenem (10 μg), ampicillin/sulbactam (220 μg), tetracycline (30 μg), and trimethoprim–sulfamethoxazole (25 μg). Susceptibility results were interpreted in accordance with *Clinical and Laboratory Standards Institute (CLSI) M100 standard, 35th edition (2025)*. Given the absence of species-specific interpretive criteria for *S. colletis*, the results were interpreted according to CLSI M100 guidelines for Enterobacterales, which may not fully reflect the true susceptibility profile of this organism.

## 3. Result

### 3.1. Genomic Features

The patient, a 64-year-old male, had an 8-year history of progressive dysuria, urinary frequency, and incomplete emptying attributed to benign prostatic hyperplasia (BPH). One month prior to the current admission, he experienced acute urinary retention and was managed with an indwelling urinary catheter. He was admitted to our department on 10 March 2025, for surgical intervention for BPH. At the time of admission, he presented with a persistent indwelling catheter and recurring symptoms of urinary tract irritation. Laboratory findings indicated a severe urinary tract infection, with urinalysis showing 60–70 WBC/HPF and quantitative urine culture of 1 × 10^7^ CFU/mL. A strain was isolated from his urine and identified as *S. colletis* by MALDI-TOF MS. This strain was subsequently subjected to whole genome sequencing for further confirmation.

A total of 8,370,834 clean reads were obtained and used for genomic assembly. The assembled genome of *S. colletis* strain S25242 was 4.19 Mb in size, with a G + C content of 57.1%. The genome coverage was 100%, with a depth of 225×. The draft genome consisted of 30 scaffolds with an N50 length of 300,771 bp. Annotation by RAST identified 4012 coding sequences that were classified into 324 subsystems (Figure S1). Additionally, 73 tRNAs, 10 rRNAs, and 38 snRNAs were identified in the genome. The category of Amino Acids and Derivatives contained the highest number of genes (n = 244), followed by genes in Carbohydrates (n = 214). Notably, genes associated with Nodulation and Photosynthesis were not detected in the genome (Figure S1). The ST of strain S25242 was identified as a novel type, with ST227 being the closest relative.

### 3.2. Phylogenetic Trees

**Figure 1:**
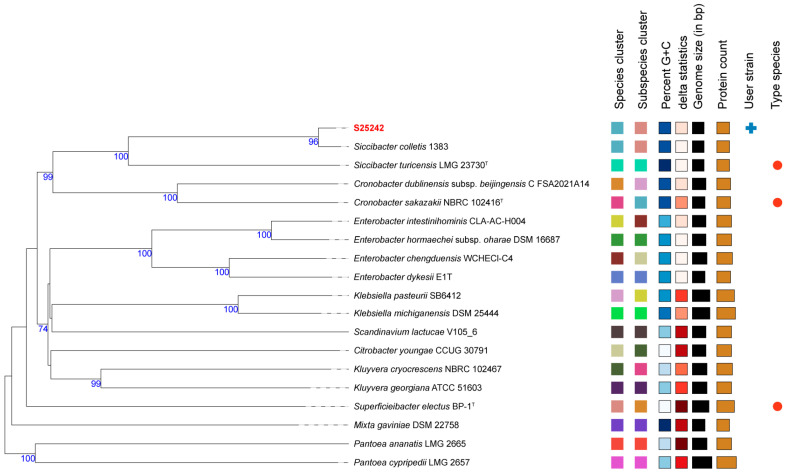
Phylogenetic tree inferred from intergenomic distances using the Type (Strain) Genome Server (TYGS). The phylogenomic tree was inferred with FastME v2.1.6.1 based on type-based species clustering with a 70% dDDH radius surrounding 18 type strains. The font for strain S25242 is in red. The right side of the figure consists of eight distinct metadata columns that characterize the strains. Species clusters (Columns 1–2): These columns use color-coded squares to represent taxonomic groupings. Strains sharing the same color belong to the same species or subspecific cluster. Genomic Characteristics: The Percent G + C and delta statistics columns use color gradients to indicate variations in these genomic markers. Quantitative Data: For Genome size (bp) and Protein count, the values are visualized as bar charts where the length of each bar is proportional to the numerical quantity. Strain Identifiers: The User strain column highlights our clinical isolate (S25242), and the Type species column identifies the reference type strains used for comparison. This detailed annotation demonstrates that S25242 shares a consistent genomic profile and clusters perfectly with the *S. colletis* lineage.

**Figure 2:**
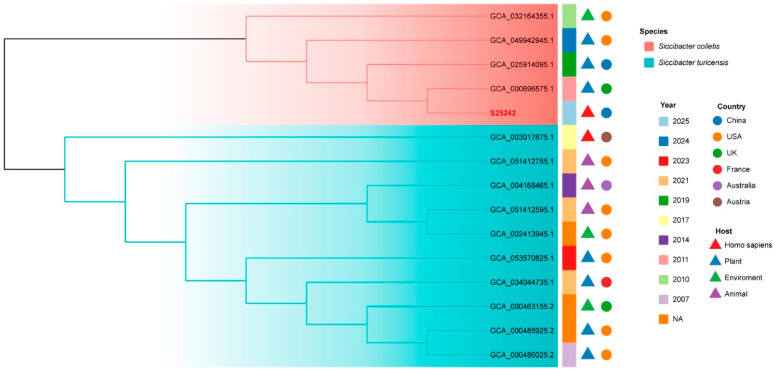
Phylogenomic tree constructed from 1186 core genes extracted from all publicly available *Siccibacter* genomes. The core genes were extracted using Roary. The branches are colored according to species. Each strain is annotated with its collected year, country of origin, and host/environment source. The strain S25242 is in a red font. NA, not available.

Phylogenetic inference was first conducted based on intergenomic distances (Figure 1), using type-based species clustering with a 70% dDDH radius around each of the 18 type strains. The results revealed that the strain S25242 fell within the genus *Siccibacter* and was closely related to the type strain of *S. colletis* strain 1383^T^. Subsequently, we downloaded all the available genomes within the genus *Siccibacter* (Table S1) and extracted their core genes. A phylogenomic tree was then constructed based on 1186 core genes. The tree (Figure 2) indicated that strain S25242 belonged to *S. colletis* and was also closely related to *S. colletis* strain 1383^T^ (GCA_000696575.1).

### 3.3. Taxonomic Confirmation

**Figure 3:**
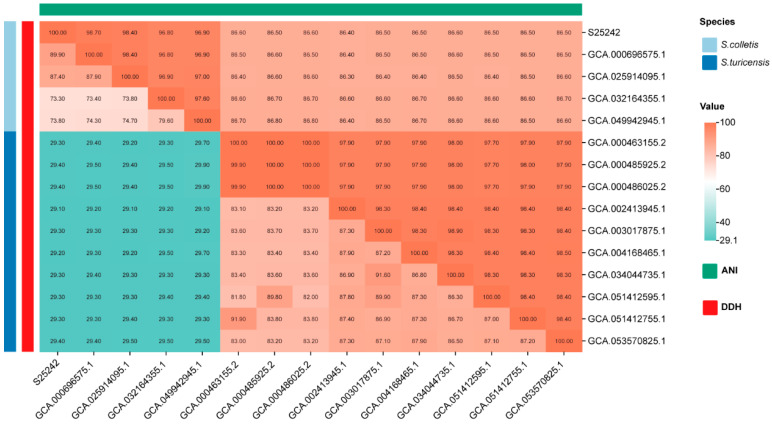
Heatmap illustrating pairwise comparisons of digital DNA-DNA hybridization (dDDH) and average nucleotide identity (ANI) across the genus *Siccibacter*. The results confirm strain S25242 belongs to *Siccibacter colletis*, showing dDDH values > 70% and ANI values > 96% with other members of the species.

To further investigate the taxonomic status, we calculated the pairwise dDDH values within the genus *Siccibacter* (Figure 3). Strain S25242 showed values greater than 70% with all members of *S. colletis* (73.8–89.9%) and values less than 30% with all members of *S. turicensis* (Figure 3). We then estimated the ANI for all genomes within the genus *Siccibacter*. The results exhibited that the pairwise values among members of *S. colletis* (including strain S25242) were higher than the 96% threshold, while the values between members of *S. colletis* and members of *S. turicensis* were less than 90%. Currently, there are four publicly available genomes for *S. colletis*: two from seeds, one from leaves, and one from cucumber fermentation brine. These findings supported that strain S25242 was the first clinical isolate of *S. colletis*.

### 3.4. Antimicrobial Susceptibility Testing Result

The results of antimicrobial susceptibility testing revealed that the inhibition zones of each antimicrobial agent varied, including imipenem (30 mm), meropenem (36 mm), ampicillin (20 mm), cefotaxime (34 mm), ceftriaxone (34 mm), sulbactam (24 mm), gentamicin (19 mm), ciprofloxacin (42 mm), levofloxacin (42 mm), trimethoprim–sulfamethoxazole (30 mm), and tetracycline (28 mm). Notably, the isolate was susceptible to all eleven antimicrobials tested, according to the CLSI guideline (Table 1). Furthermore, we did not detect any ARGs in strain S25242, which was consistent with the above phenotypic findings.

### 3.5. Genomic Comparison

A comparative genomics study was conducted on all the available genomes within the genus *Siccibacter* (Table S1). A total of 9095 genes were identified, among which 1942 (21.4%, 1942/9095) were unique to members of *S. colletis* (Figure S2). Annotation of these unique genes revealed that 24.0% (467/1942) encoded hypothetical proteins, while 76.0% (1475/1942) encoded functional proteins. The functional proteins were associated with drug resistance (multidrug efflux proteins: AcrE and AcrF), transcriptional regulation (HTH-type transcriptional regulatory factors: DmlR, PgrR, and NorR), substance transport (proline/betaine transporter, branched-chain amino acid transport ATP-binding protein LivF), metabolism (*β*-phosphogluconate mutase, α subunit of fatty acid oxidation complex), and signal transduction (Methyl-accepting chemotaxis protein).

**Figure 4:**
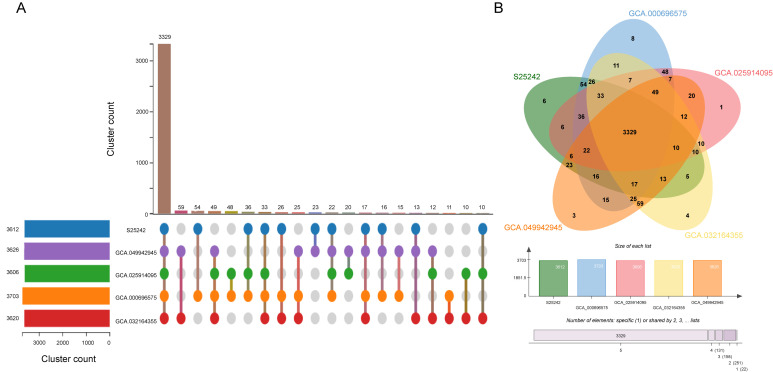
Comparative genomics of five *Siccibacter colletis* strains. Orthologous gene cluster analysis among *Siccibacter colletis* genomes was performed using OrthoVenn3. (**A**) An UpSet plot visualizes the intersection of gene clusters across the genomes, highlighting 3329 core orthologous clusters shared by all five strains. (**B**) A Venn diagram displays the distribution of shared and unique clusters, including the six unique clusters (13 genes) identified exclusively in the clinical strain S25242.

Subsequently, comparative analysis among genomes within the *S. colletis* species was further investigated using OrthoVenn3 (Figure 4). The results showed that 3612, 3703, 3626, 3606, and 3620 clusters were detected in S25242, GCA_000696575, GCA_049942945, GCA_025914095, and GCA_032164355, respectively (Figure S3). Among these, 3891 orthologous clusters were identified, of which 3329 were shared among all five genomes (Figure 4). Notably, six orthologous clusters comprising 13 genes were identified exclusively in strain S25242. Gene ontology (GO) annotation of these genes showed that they were involved in transposition, DNA integration, regulation of transcription, metal ion binding, and phosphorelay sensor kinase activity (Table S2). Detailed structural mapping revealed that eight of the unique genes in strain S25242 are embedded within a larger genomic block flanked by insertion sequences (Figure S4). This region exhibits a mosaic structure, containing both unique genes and conserved sequences common to other *S. colletis* strains. Such a configuration is characteristic of genomic islands (GIs), where a complex suite of genes is acquired through horizontal gene transfer. The integration of this mobile element likely provided S25242 with novel functional capabilities while maintaining essential genomic architecture. Although the assembly of our draft genome prevents the full physical linkage analysis of the remaining five unique genes, the presence of this IS-flanked cluster underscores the significant role of genomic plasticity and recombination in the adaptation of this clinical isolate.

## 4. Discussion

The genus *Siccibacter* constitutes a relatively recently defined group within the family *Enterobacteriaceae* [1,2]. Distinguished by its exceptional desiccation resistance, this genus has garnered increasing scrutiny in food safety and clinical microbiology, primarily due to its close phylogenetic proximity to the neonatal pathogen *Cronobacter* [32,33]. Among its members, *S. colletis* has traditionally been recognized as an environmental species, frequently isolated from plant-based matrices such as poppy seeds and tea leaves [5]. In this study, we reported the first clinical isolation of *S. colletis* (strain S25242), recovered from a severe urinary tract infection. It is crucial to distinguish true infection from transient colonization, especially for environmental species. In this case, the high bacterial load in the urine culture (1 × 10^7^ CFU/mL), coupled with significant pyuria (60–70 WBC/HPF) and the patient’s symptomatic presentation, strongly indicates that *S. colletis* was the primary causative agent of the urinary tract infection rather than a transient inhabitant. To our knowledge, this study represents the first clinical report of *S. colletis* isolated from a human specimen. While the species is present in some MALDI-TOF MS databases (e.g., Bruker Biotyper), its clinical recognition has been hindered by its rarity in medical settings. Our findings underscore the importance of expanding proteomic databases to include emerging opportunistic pathogens that were previously considered purely environmental.

We sequenced the whole genome and investigated the genomic characteristics of strain S25242. Genomes of *Siccibacter* species typically range from 4.1 to 4.3 Mb, with a G + C content of approximately 57.0–58.5% (https://www.ncbi.nlm.nih.gov/datasets/genome/?taxon=1649298 (accessed on 16 February 2026)). The genomic size of strain S25242 was 4.19 Mb, with a G + C content of 57.1%, both of which fall within this type range. Phylogenetic and phylogenomic analyses indicated that S25242 is most closely related to the type strain *S. colletis* 1383^T^. Furthermore, the ANI and DDH values between strain S25242 and the type strain *S. colletis* 1383^T^ were higher than the commonly accepted thresholds (70% for DDH and 96% for ANI) [24,25]. These results support the identification of strain S25242 as the first clinical isolate of *S. colletis*.

Our comparative genomic analysis conducted in this study provides the first comprehensive blueprint of the genetic architecture of *S. colletis*, particularly highlighting the features that distinguish it from other members of the *Siccibacter* genus. Our finding that 21.4% (1942/9095) of the total gene pool is unique to *S. colletis* suggests a significant degree of species-specific evolution, potentially driven by the need to adapt to diverse ecological niches, including the clinical environment. The identification of multidrug efflux systems (AcrE and AcrF) and regulators like NorR underscores a robust capacity for antimicrobial resistance and stress response, which are essential for clinical persistence [34,35]. These findings are particularly noteworthy given that clinical infections by *Siccibacter* species remain infrequently reported in the literature [7].

Intra-species comparison highlights a stable core genome of 3329 clusters, yet the clinical strain S25242 harbors unique genetic signatures. The 13 genes exclusive to S25242, involved in transposition and DNA integration, point toward recent horizontal gene transfer events [36], a hallmark of pathogen evolution in hospital environments. Furthermore, the presence of phosphorelay sensor kinases and metal ion-binding proteins suggests sophisticated machinery for sensing host signals and nutrient acquisition, both of which are critical factors for opportunistic infection.

Interestingly, virulence factor analysis via VFDB identified the presence of the outer membrane protein A (*ompA*) in strain S25242. OmpA is known to contribute significantly to the pathogenesis of *Enterobacteriaceae* by facilitating adhesion to epithelial cells and contributing to biofilm formation, which are critical steps in establishing urinary tract infections. The lack of potent toxins, combined with the presence of *ompA* and host predisposing factors, further reinforces the classification of *S. colletis* as an opportunistic pathogen rather than a primary one. This microevolutionary process underscores the potential of environmental *Siccibacter* species to cross the barrier into clinical relevance under suitable host conditions.

The identification of *S. colletis* in a severe clinical infection carries significant public health implications. As the boundaries between environmental and clinical microbiomes continue to blur, adding such emerging species to standard clinical screening databases is essential for accurate diagnosis and for monitoring the global spread of opportunistic pathogens. Standardizing the screening of *S. colletis* allows public health authorities to track the “spillover” of bacteria from environmental/plant sources into the healthcare system. Given the genomic plasticity and latent resistance potential identified in this study (e.g., AcrE and AcrF efflux systems), early identification is crucial for effective infection control and monitoring the evolution of antimicrobial resistance in rare pathogens.

A key limitation of this study lies in the antimicrobial susceptibility testing strategy. As *S. colletis* is a rarely reported species without established clinical breakpoints, the use of disk diffusion and interpretation based on CLSI criteria for Enterobacterales may not accurately represent its true susceptibility profile. Broth microdilution is generally considered the reference method for antimicrobial susceptibility testing, particularly for uncommon or newly described organisms. Therefore, the results presented here should be regarded as descriptive and interpreted with caution, rather than definitive for clinical decision making. Notably, no acquired antimicrobial resistance genes were identified in the genome, which is concordant with the phenotypic findings and partially supports the observed susceptibility profile.

## 5. Conclusions

In conclusion, prior to this report, clinical infections associated with *Siccibacter* were exceedingly rare, with only one documented case involving *S. turicensis* [7]. The identification of strain S25242 in a clinical context represents a significant paradigm shift, challenging the previous perception of this species as purely environmental. Our findings indicate that while *S. colletis* maintains a conserved core, the acquisition of mobile genetic elements and specialized sensory systems facilitates its emergence as an opportunistic pathogen. This genomic plasticity, coupled with its latent resistance potential, highlights the need for increased surveillance of the *Siccibacter* genus to prevent unrecognized infections.

## Figures and Tables

**Table 1:** The antimicrobial susceptibility testing result of strain S25242 from this study.

Antimicrobial Agents (Dosage)	Zone Diameter (mm)	Susceptibility	CLSI Standards		
			S	I	R
Imipenem (10 μg)	30	S	≥23	20–22	≤19
Meropenem (10 μg)	36	S	≥23	20–22	≤19
Ampicillin (10 μg)	20	S	≥17	14–16	≤13
Cefotaxime (30 μg)	34	S	≥26	23–25	≤22
Ceftriaxone (30 μg)	34	S	≥23	20–22	≤19
Ampicillin/sulbactam (20 μg)	24	S	≥15	12–14	≤11
Gentamicin (10 μg)	19	S	≥18	15–17	≤14
Ciprofloxacin (5 μg)	42	S	≥26	22–25	≤21
Levofloxacin (5 μg)	42	S	≥21	17–20	≤16
Trimethoprim–sulfamethoxazole (25 μg)	30	S	≥16	11–15	≤10
Tetracycline (30 μg)	28	S	≥15	12–14	≤11

S: susceptibility, I: intermediate, R: resistant. CLSI: Clinical and Laboratory Standards Institute. Trimethoprim–sulfamethoxazole (25 μg): trimethoprim–sulfamethoxazole (1.25/23.75 μg).

## Data Availability

The genomes from this study are openly available in NCBI database under accession number PRJNA1414882.

## Supplementary Materials

The following supporting information can be downloaded at: https://www.mdpi.com/article/10.3390/microorganisms14040932/s1, Table S1: Genomic information of available *Siccibacter* spp. Table S2: Unique clusters identified in strain S25242. Figure S1: RAST functional annotation of *Siccibacter colletis* strain S25242, displaying the distribution of 4012 encoding sequences across 324 subsystems. Figure S2: Comparative genomic analysis of all available *Siccibacter* genomes, highlighting 1942 genes unique to the *Siccibacter colletis* species. Figure S3: Detailed OrthoVenn3 cluster detection results for each of the five *S. colletis* genomes. Figure S4: The gene location of unique genes identified in this study. The unique genes were labeled with red colors.
