# Supplementary material for: Genomic Evolution of Siccibacter colletis: Comparative Analysis and First Clinical Isolate Report
Source: Microorganisms. 2026 Apr 20;14(4):932. doi: 10.3390/microorganisms14040932 (PMC13118582; doi:10.3390/microorganisms14040932)
Supplement: Supplementary file 1 [file microorganisms-14-00932-s001.zip › microorganisms-4235642-supplementary.pdf]

Supplementary materials

Genomic Evolution of *Siccibacter colletis*: Comparative Analysis and First Clinical Isolate Report

Table S1. Genomic information of available *Siccibacter* spp.

ID	Species	Host	Year	Country
S25242	*S. colletis*	Homo sapiens	2025	China
GCA_049942945.1	*S. colletis*	Plant	2024	USA
GCA_032164355.1	*S. colletis*	Environment	2010	USA
GCA_025914095.1	*S. colletis*	Plant	2019	China
GCA_000696575.1	*S. colletis*	Plant	2011	UK
GCA_053570825.1	*S. turicensis*	Plant	2023	USA
GCA_051412595.1	*S. turicensis*	Animal	2021	USA
GCA_051412755.1	*S. turicensis*	Animal	2021	USA
GCA_000485925.2	*S. turicensis*	Plant	NA	USA
GCA_034044735.1	*S. turicensis*	Plant	2021	France
GCA_000486025.2	*S. turicensis*	Plant	2007	USA
GCA_004168465.1	*S. turicensis*	Animal	2014	Australia
GCA_003017875.1	*S. turicensis*	Homo sapiens	2017	Austria
GCA_002413945.1	*S. turicensis*	Environment	NA	USA
GCA_000463155.2	*S. turicensis*	Environment	NA	UK

Table S2. Unique clusters identified in strain S25242.

Cluster ID	Number Of proteins	Swiss-Prot Hit	GO Annotation	Function
cluster3473	3	P0CF79	GO:0032196; P:transposition; IDA:EcoCyc	Transposase InsF for insertion sequence IS3A
cluster3635	2	Q52563	GO:0015074; P:DNA integration; IEA:UniProtKB-KW	Resolvase
cluster3636	2	Q9ZHD3	GO:0006355; P:regulation of transcription, DNA-templated; IEA:InterPro	Probable transcriptional regulatory protein
cluster3637	2	Q9Z4N3	GO:0046872; F:metal ion binding; IEA:UniProtKB-KW	Silver-binding protein SilE
cluster3638	2	Q47457	GO:0000155; F:phosphorelay sensor kinase activity; IEA:InterPro	Probable sensor protein PcoS
cluster3639	2	N/A	N/A	N/A

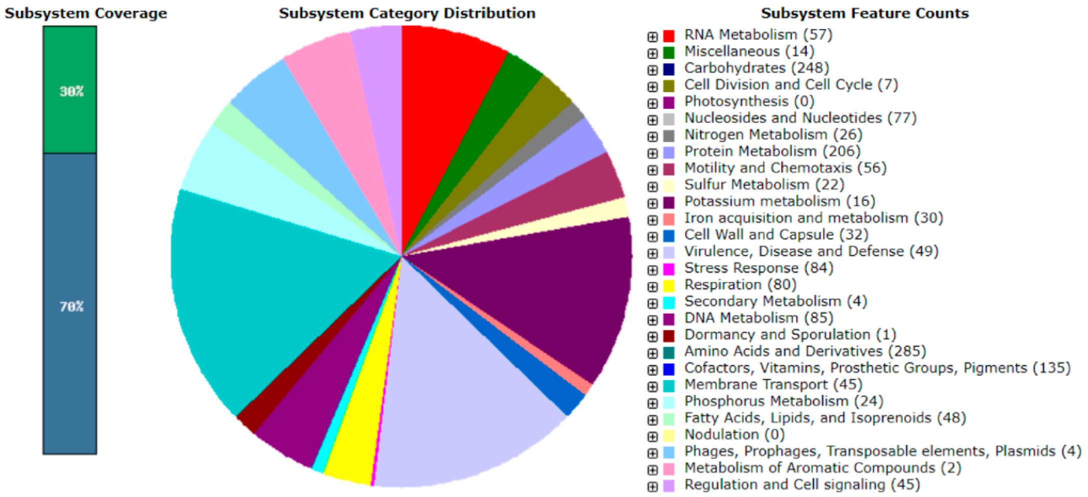

Figure S1. RAST functional annotation of *Siccibacter colletis* strain S25242, displaying the distribution of 4,012 encoding sequences across 324 subsystems.

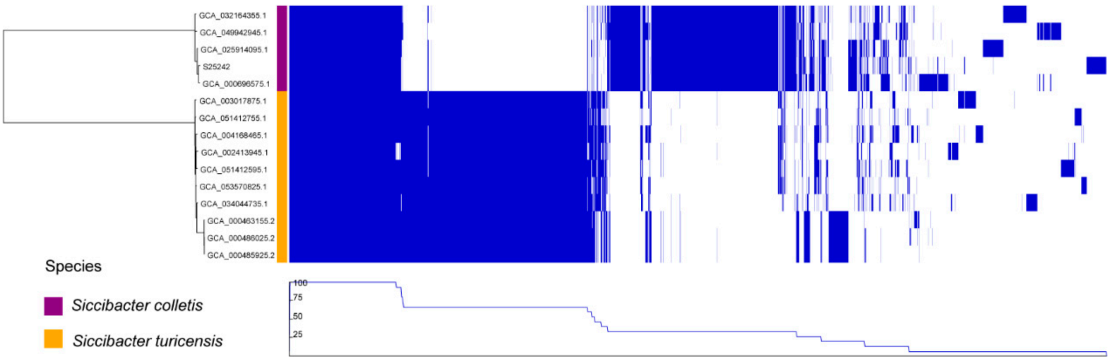

Figure S2. Comparative genomic analysis of all available *Siccibacter* genomes, highlighting 1,942 genes unique to the *Siccibacter colletis* species.

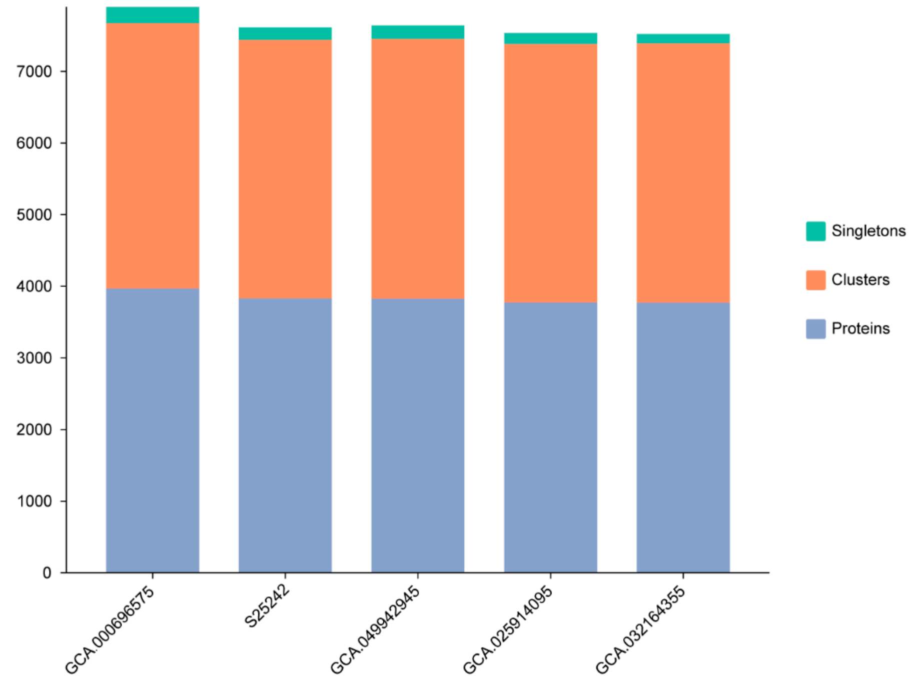

Figure S3. Detailed OrthoVenn3 cluster detection results for each of the five *S. colletis* genomes.

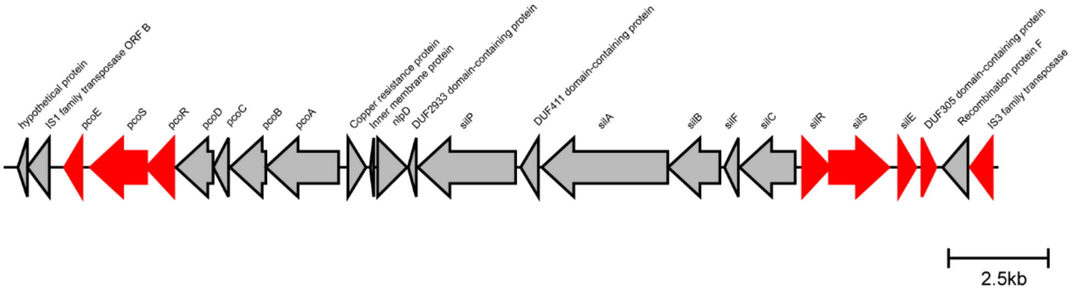

Figure S4. The gene location of unique genes identified in this study. The unique genes were labeled with red colors.
